# Dietary *Macleaya cordata* extract supplementation improves the growth performance and gut health of broiler chickens with necrotic enteritis

**DOI:** 10.1186/s40104-023-00916-2

**Published:** 2023-09-07

**Authors:** Bochen Song, Jie He, Xue Pan, Linglian Kong, Chuanpi Xiao, Chake Keerqin, Zhigang Song

**Affiliations:** 1 Key Laboratory of Efficient Utilization of Non-Grain Feed Resources, College of Animal Science and Technology, Shandong Agricultural University, Taian, 271018 Shandong China; 2 Center for Mitochondria and Healthy Ageing, College of Life Sciences, Yantai University, Yantai, 264005 Shandong China; 3 Precision Livestock and Nutrition Unit, University of Liège, Gembloux Agro-Bio TechGembloux, Belgium; 4 Phytobiotics (Jiangsu) Biotech Co., Ltd., Jintan, 213200 China

**Keywords:** Broiler chicken, Gut microbiota, Immune function, *Macleayacordata* extract, Necrotic enteritis

## Abstract

**Background:**

The poultry industry needs effective antibiotic alternatives to control outbreaks of necrotic enteritis (NE) caused by *Clostridium perfringens*.

**Methods:**

The aim of this study was to investigate the effects of dietary supplementation with *Macleaya cordata* extract (MCE) on the immune function and gut microbiota of broilers with NE. A total of 288 1-day-old broiler chicks were randomly assigned to a 2 × 2 factorial arrangement with two concentrations of dietary MCE supplementation (0 or 350 mg/kg of diet) and two disease challenge statuses (control or NE).

**Results:**

The results revealed that NE significantly increased the feed conversion rate (FCR), mortality, intestinal lesion score, the levels of IL-1β, IL-17 and IFN-γ/IL-4 in serum and IL-17/IL-10 in the jejunal mucosa, mRNA levels of *TLR2*, *IFN-γ* and *pIgR* in the jejunum, and *Clostridium perfringens* concentrations in the cecum. NE significantly decreased the body weight (BW), body weight gain (BWG), jejunal villus height, V/C, mRNA level of *AMPK-α1* in jejunum, IL-4 level in the jejunal mucosa and lactic acid bacteria abundance in the cecum. MCE significantly increased BW, BWG, jejunal villus height, V/C, mRNA levels of *occludin*, *ZO-1* and *AMPK-α1* in the jejunum, the levels of IgA and IgG in serum and IL-10 in the jejunal mucosa and mRNA levels of *NF-κB*, *IL-10* and *MHC-II* in the jejunum. Additionally, MCE significantly decreased the FCR, mortality, intestinal lesion score, jejunal crypt depth, the levels of IFN-γ and IL-17 in serum and IL-17/IL-10 in the jejunal mucosa, *Clostridium perfringens* concentrations in the cecum, and mRNA levels of *IL-17/IL-10* in the jejunum. Moreover, NE significantly increased the abundance of bacteria that are associated with inflammation, obesity and depression (*Alistipes*, *Barnesiella*, *Intestinimonas*, *RF39* and *UCG-005*) and significantly decreased the abundance of short-chain fatty acid (SCFA)-producing bacteria (*Anaerotruncus*, *Butyricicoccus* and *Bacteroides*) in the cecum. MCE significantly increased the abundance of SCFA-producing bacteria (*Streptococcus*, *Ruminococcus_torques_group* and *Lachnospiraceae_NK4A136_group*) and significantly reduced the abundance of bacteria that are associated with inflammation and obesity (*Alistipes*, *Barnesiella* and *UCG-010*) in the cecum. In the cecum of broilers with NE, the relative abundance of *Barnesiella* and *Alistipes* was higher and that of *Lachnoclostridium* and *Shuttleworthia* was lower. Interestingly, these trends were reversed by the addition of MCE to the diet. Spearman correlation analysis showed that *Barnesiella* and *Alistipes* were associated with enhanced intestinal inflammation and inhibited growth performance, whereas *Lachnoclostridium* and *Shuttleworthia* were associated with anti-inflammatory effects.

**Conclusions:**

MCE ameliorated the loss of growth performance in broiler chickens with NE, probably by regulating the intestinal barrier, immune function, and gut microbiota.

**Supplementary Information:**

The online version contains supplementary material available at 10.1186/s40104-023-00916-2.

## Introduction

Necrotic enteritis (NE) is an enterotoxemic disease in poultry that is caused by *Clostridium perfringens*. The ban on the addition of antibiotics to feed in locations such as the EU and China has led to an increase in the incidence of NE [1]. NE in chickens is relatively lethal, with mortality rates of up to 50% in acute cases, especially in young chickens. During NE, the levels of pro-inflammatory cytokines and chemokines in chickens dramatically increase [2]. Since inflammation is an energy-consuming process, it has been estimated that animals increase their resting metabolic rate by 8% to 27% during immune stress [3]. Inflammation reduces feed consumption, disrupts the morphological structure of the gut, impairs nutrient absorption, and alters anabolic processes in skeletal muscle so that nutrients can be used for immune function [4, 5]. As a result, chronic NE significantly reduces the growth performance of chickens, causing intestinal ulceration and erosion, which seriously affects the health of poultry and causes serious economic losses in poultry farming [6].

*Macleaya cordata* extract (MCE) is a perennial herb and a Chinese herbal medicine that is widely distributed in southern China. Compounds containing sanguinarine and chelerythrine were registered as feed additives in the EU in 2004. Sanguinarine that is isolated from *Macleaya cordata* is the primary bioactive substance of this plant. Sanguinarine exerts antitumor [7], immune-enhancing [8], antibacterial [9], anti-inflammatory [10] and insecticidal [11] effects. In recent years, MCE has been added to the diets of chickens, pigs, cattle and fish. Dietary MCE supplementation improved the growth performance [12] and intestinal barrier [13] of broilers and exerted anti-inflammatory effects [14]. It was reported that dietary supplementation with 0.6 mg/kg MCE significantly increased the average daily weight gain and significantly reduced the feed conversion rate (FCR) of broilers from d 0 to 42 [12]. Dietary supplementation with 100 mg/kg MCE significantly improved growth performance, reduced lipid peroxide, corticosterone, uric acid and FITC-D levels in serum, and reduced the mRNA levels of *IL-6*, *TNF-α* and *iNOS* in the ileum of heat-stressed broilers [13]. An oral solution containing 1% MCE significantly increased body weight gain (BWG), serum IgG levels and jejunum villus height and decreased serum IL-1β levels in broilers [14]. Dietary supplementation with 120 mg/kg MCE significantly increased growth performance and decreased intestinal lesion scores of broilers with NE [15]. Dietary supplementation with 150 mg/kg MCE significantly increased feed intake (FI), BWG and breast meat percentage and decreased lesion scores in the duodenum, jejunum and ileum of broilers with NE [10]. In addition to the loss of growth performance, dysbiosis of the gut microbiota is a key characteristic of intestinal inflammation. Dysregulation of the microbiota is characterized by altered composition, reduced diversity and stability, and increased abundance of lipopolysaccharide-carrying pro-inflammatory bacteria [16].

The gut microbiota is the largest symbiotic ecosystem in hosts and has been shown to play an important role in maintaining intestinal homeostasis. Changes in the gut microbiota can confer resistance to pathogenic bacteria or promote infection in a host. A symbiotic microbiome regulates the maturation of the mucosal immune system, while a pathogenic microbiome can cause immune dysfunction in the host, leading to the development of diseases such as intestinal inflammation [17]. Pathogenic bacteria use microbiota-derived carbon and nitrogen sources as nutrients and regulatory signals to promote their own growth and virulence. By inducing inflammation, these bacteria alter the gut environment and use a unique respiratory and metal acquisition system to drive their expansion [18].

Dietary MCE supplementation has been reported to primarily alter the microbiota of the front half of the intestine of chickens, promoting the proliferation of *Lactobacillus*, inhibiting the colonization of *Escherichia coli*, and activating amino acid, vitamin and secondary bile acid biosynthetic pathways while avoiding the accumulation of antibiotic resistance genes [19]. Dietary supplementation with 100 mg/kg MCE significantly increased the diversity of the microbiota in the ileum of Snowy Peak blackbone chickens, and 200 mg/kg MCE significantly increased the relative abundance of *Lactobacillus* and *Aeriscardovia* in the ileum, increased the relative abundance of *Bacteroidetes* and *Deferribacteres* in the cecum, and decreased the relative abundance of Firmicutes in the cecum [20].

Previous studies have shown that MCE improves growth performance and intestinal barrier function and ameliorates intestinal inflammation in broilers. However, there is still a lack of evidence about the effect of MCE on humoral immune function and the ability of MCE to ameliorate NE in broiler chickens via regulation of the gut microbiota. In this study, we investigated the effects of MCE on the growth performance, intestinal barrier function, immune function, and gut microbiota of broilers with NE by establishing a model of NE via coinfection with *Clostridium perfringens* and coccidia. We aimed to investigate the mechanism by which MCE alleviates NE in broilers by regulating the gut microbiota and to reveal key microbes that are associated with the effect of MCE in ameliorating NE in broilers.

## Materials and methods

### Experimental design, broilers and diets

A 2 × 2 factorial arrangement of treatments was employed in a completely randomized design to investigate the effects of two concentrations of *Macleaya cordata* extract (0 or 350 mg/kg of diet) and two NE challenge statuses (challenged or unchallenged). A total of 288 1-day-old male broiler chicks were obtained from a commercial hatchery (Liaocheng Hekangyuan Animal Husbandry Co., Ltd., Liaocheng, China). Upon arrival, the broilers were weighed after hatching and randomly assigned to one of the four treatment groups. Each treatment group had 6 replicate cages with 12 broilers per cage. The treatment groups were as follows: (i) NC, negative control group (neither MCE treatment nor NE); (ii) MCE, MCE-treated group (MCE treatment without NE); (iii) PC, NE control group (NE without MCE treatment); and (iv) NEMCE, MCE-treated and NE group (both MCE treatment and NE). The active ingredient of the feed additive that was used in this study was isoquinoline alkaloids (at least 0.375%, containing at least 0.15% sanguinarine). The ingredients and nutrient levels in the basal diet (Table 1) were formulated according to standards of the National Research Council (NRC, 1994) [21]. All broilers were weighed and randomly assigned to 24 metal cages (70 cm × 70 cm × 40 cm), which were equipped with feeders and nipple drinkers. Broilers with similar initial weight were reared in a room with a controlled environment. Each group included 6 replicate cages with 12 broilers per cage. The initial temperature was 35 °C, and then, the temperature was gradually decreased to 25 °C by 30 days of age. The average relative humidity was maintained at approximately 70% in the first 3 d and thereafter maintained between 55% and 65%. Broilers were kept under 23 h of light and 1 h of darkness in the first week, followed by 20 h of light and 4 h of darkness for the subsequent period.

**Table 1:** Ingredients and composition (calculated and analyzed nutrients) of the experimental diets^1^ (%, unless otherwise noted, as-fed basis)

Item	d 1 to 21	d 22 to 30
Composition, %		
Corn (7.8% CP)	51.38	60.02
Soybean meal (46% CP)	40.71	25.54
Corn protein flour	0.00	5.66
Soybean oil	3.75	3.32
Wheat flour	0.00	2.00
CaHPO_3_·2H_2_O	1.86	1.33
Stone powder (37%)	1.24	1.14
Sodium chloride	0.35	0.35
*DL*-Methionine (98%)	0.20	0.070
*L*-Lysine HCl (98%)	0.00	0.19
Vitamin premix^2^	0.03	0.03
Mineral premix^3^	0.20	0.20
Choline chloride (50%)	0.25	0.16
Sandoquin (Ethoxyquinoline)	0.030	0.00
Calculated nutrient levels^4^		
Metabolizable energy, kcal/kg	2,928.97	3,100.00
Crude protein	21.76	20.00
Calcium	1.01	0.90
Available phosphorus	0.44	0.35
Lysine	1.14	1.00
Methionine	0.54	0.40

^1^Diets were in mash form

^2^Vitamin premix provided per kg of complete diet: vitamin A (retinylacetate), 9,500 IU; vitamin D_3_ (cholecalciferol), 2,500 IU; vitamin E (*DL*-α-tocopherol acetate), 30 IU; vitamin K_3_ (menadione sodium bisulfate), 2.65 mg; vitamin B_12_ (cyanocobalamin), 0.025 mg; biotin, 0.30 mg; folic acid, 1.25 mg; nicotinic acid, 50 mg; *D*-pantothenic acid, 12 mg; pyridoxine hydrochloride, 6.0 mg; riboflavin, 6.5 mg; thiamine mononitrate, 3.0 mg

^3^Mineral premix provided per kg of complete diet: iron, 80 mg; copper, 8 mg; manganese, 100 mg; zinc, 80 mg; iodine, 0.35 mg; selenium, 0.15 mg

^4^Calculated value based on the analysis of experimental diets

### Establishment of the NE model

*C. perfringens* type A CVCC2030 (China Veterinary Culture Collection Center, China Institute of Veterinary Drug Control, Beijing, China) was stored in fluid thioglycollate medium (CM801; Beijing Land Bridge Technology Co., Ltd., China) supplemented with 30% (v/v) glycerol at −80 °C until further use. Approximately 1 mL of the seed stock was cultured in 250 mL of thioglycollate broth for approximately 24 h at 37 °C; then, 1 mL of this culture was inoculated into 1 L of thioglycollate broth that was supplemented with 10 g/L starch and 15 g/L peptone and incubated for approximately 24 h at 37 °C.

Subclinical NE was induced in the broilers as previously described with minor modifications [22]. On d 13, each bird in the challenge groups was orally inoculated with a 30-fold dose of attenuated coccidial vaccine (containing live attenuated oocysts of *Eimeria tenella* PTMZ strain, *E. necatrix* PNHZ strain*, E. maxima* PMHY strain, and *E. acervulina* PAHY strain; Foshan Standard Bio-Tech Co., Ltd., Foshan, China). Uninfected control broilers received 1 mL of sterile PBS instead of the vaccine. All the *Eimeria* oocyst-inoculated broilers were subsequently orally gavaged with 1 mL of *C. perfringens* (1 × 10^9^ CFU/mL) per day from d 17 to 23. The uninfected broilers were orally gavaged with 1 mL of sterile thioglycollate broth at the same time points. Feed was withdrawn 8 h prior to each inoculation.

### Sample collection and index determination

#### Growth performance

On d 13, 24 and 30, feed consumption and body weight in each replicate were recorded. BWG and FI were subsequently calculated. Spilled feed was carefully collected and weighed to correct the final FI data. The FCR was defined as FI:BWG. Mortality data were recorded and included in the FCR calculation.

#### Intestinal lesion score

On d 30, the intestinal lesion score was determined according to a previously described method; the lesions were observed, and the lesion scores were evaluated [23].

#### Intestinal tissue morphology

Fixed intestinal tissues were dehydrated and embedded in paraffin. Tissue sections (thickness of 4 μm) were stained with hematoxylin and eosin (HE, Olympus BX50; Tokyo, Japan). Ten intact intestinal villi were randomly selected in each slice and observed by a Leica microscope (Wetzlar, Germany, Model DMi8). Image-ProPlus (version 6.0) software was used to measure the height of each intestinal villus and its corresponding crypt depth and to calculate the ratio of the two values. The height of the villi was defined as the vertical distance from the tip of the villi to the villi-crypt junction, and the crypt depth was defined as the vertical distance from the villi-crypt junction to the base of the crypt.

#### Serum and jejunal mucosal concentrations of cytokines and immunoglobulins

Serum concentrations of interleukin 1 beta (IL-1β), interleukin 4 (IL-4), interleukin 10 (IL-10), interleukin 17 (IL-17), interferon-gamma (IFN-γ), immunoglobulin A (IgA), and immunoglobulin G (IgG) were measured using enzyme-linked immunosorbent assay (ELISA) kits (mlbio, Shanghai, China). Jejunum samples (0.3 g) were homogenized in 2.7 mL of phosphate-buffered saline and centrifuged at 1,000 × *g* at 4 °C for 10 min. Then, the supernatants were collected to measure the concentrations of IL-1β, IL-4, IL-10, IL-17, IFN-γ, secretory immunoglobulin A (sIgA), and IgG by ELISAs (mlbio, Shanghai, China). The results were normalized to the protein concentration in each jejunal homogenate. All determination procedures were performed strictly according to the manufacturer's instructions. The inter- and intra-assay coefficients of variation (CV) were less than 10%.

#### Quantitative real-time PCR analysis

On d 30, molecular samples of the jejunum were quick-frozen in liquid nitrogen and then transferred to a −80 °C low-temperature freezer; subsequently, these samples were to measure the expression levels of immune function-related genes in the jejunum. TRIzol reagent was used to extract total jejunum RNA, and a NanoDrop ultra-micro-calculation protein analyzer was used to determine the RNA quality and concentration. The reagent kit that was used in the reverse transcription step was the PrimeScript™ RT Reagent Kit with gDNA Eraser (Perfect Real Time) from Takara. The cDNA obtained after reverse transcription was subjected to real-time fluorescent quantitative PCR on an ABI 7500 real-time fluorescent quantitative PCR instrument with the primers that are listed in Table 2. The fluorescence quantification kit was Takara SYBR® Premix Ex Taq™ II (Tli RNaseH Plus), with GAPDH as the internal reference, and the results were calculated with the following formula: 2^−^^△△CT^.

**Table 2:** Sequences of the oligonucleotide primers used for quantitative real-time PCR^1^

Gene^2^	Primer sequence^3 ^(5′→3′)	GenBank accession NO.
*AMPK-α1*	F: CGGAGATAAAACAGAAGCACGAG	DQ302133
	R: CGATTCAGGATCTTCACTGCAAC	
*Claudin-1*	F: AAGTGCATGGAGGATGACCA	NM_001013611.2
	R: GCCACTCTGTTGCCATACCA	
*GAPDH*	F: AGAACATCATCCCAGCGTCC	NM_204305
	R: CGGCAGGTCAGGTCAACAAC	
*IFN-γ*	F: AAAGCCGCACATCAAACACA	NM_205149.1
	R: GCCATCAGGAAGGTTGTTTTTC	
*IgA*	F: ACCACGGCTCTGACTGTACC	S40610.1
	R: CGATGGTCTCCTTCACATCA	
*IL-1β*	F: TGGGCATCAAGGGCTACA	NM_204524.1
	R: CGGCCCACGTAGTAAATGAT	
*IL-4*	F: GTGCCCACGCTGTGCTTAC	NM_001007079.1
	R: AGGAAACCTCTCCCTGGATGTC	
*IL-10*	F: CGCTGTCACCGCTTCTTCA	AJ621614
	R: TCCCGTTCTCATCCATCTTCTC	
*IL-17*	F: CTCCGATCCCTTATTCTCCTC	AJ493595
	R: AAGCGGTTGTGGTCCTCAT	
*MHC-II*	F: CCACGGACGTGATGCAGAAC	113,206,149
	R: ACCGCGCAGGAACACGAAGA	
*NF-κB*	F: TGGAGAAGGCTATGCAGCTT	NM_205134.1
	R: CATCCTGGACAGCAGTGAGA	
*Occludin*	F: AGTTCGACACCGACCTGAAG	NM_205128.1
	R: TCCTGGTATTGAGGGCTGTC	
*pIgR*	F: ATTTGTCACCACCACAGCCA	NM_001044644
	R: GAGTAGGCGAGGTCAGCATC	
*TLR2*	F: ACCTTCTGCACTCTGCCATT	NM_204278.1
	R: TGTGAATGAAGCACCGGTAA	
*TLR4*	F: GATGCATCCCCAGTCCGTG	NM_001030693
	R: CCAGGGTGGTGTTTGGGATT	
*ZO-1*	F: ACAGCTCATCACAGCCTCCT	XM_015278981.1
	R: TGAAGGGCTTACAGGAATGG	

^1^Primers were designed using Primer Express software (Sangon Biotech, Shanghai, China)

^2^Abbreviations: *AMPK-α1* Adenosine 5'-monophosphate (AMP)-activated protein kinase-α1, *GAPDH* Glyceraldehyde-3-phosphate dehydrogenase, *IFN-γ* Interferon-γ, *IgA* Immune globulin A, *IL-1β* Interleukin-1β, *MHC-II* Major histocompatibility complex class 2, *NF-κB* Nuclear factor kappa-β, pIgR Polymeric immunoglobulin receptor, *TLR2* Toll-like receptor 2, *ZO-1* Zonula occludens-1

^3^*F* Forward, *R* Reverse

#### Abundance of Clostridium perfringens and lactic acid bacteria in the cecum

Cecum specimens were collected from broiler chickens on d 30 under aseptic conditions, quickly frozen in liquid nitrogen and stored at −20 °C to determine cecal bacterial counts. The specific method was as follows. The cecum was placed on ice (approximately 4 °C) to thaw, and 0.3 g was weighed on a balance on a clean bench and placed in a 5-mL sterile centrifuge tube. Then, 2.7 mL of sterile saline was added to achieve a tenfold dilution, and the sample was shaken and mixed on a micro shaker and allowed to stand for 10 min. Then, 0.3 mL of the supernatant was moved to a sterile centrifuge tube, and sterile saline was used to perform gradient dilutions of 10^2^, 10^3^, 10^4^, 10^5^, 10^6^, 10^7^ and 10^8^. One hundred microliters of each dilution were inoculated on the corresponding selective medium, and the bacteria were spread on the plate until the solution on the medium was dry. After culturing under the corresponding conditions, 30–300 colonies were selected to determine bacterial counts. Among them, *Clostridium perfringens* was selected on tryptone-sulfite-cycloserine (TSC) medium (CM138, Beijing Luqiao Technology Co., Ltd., Beijing, China) and cultured under anaerobic conditions at 37 °C. The colonies on the plates were counted after 24 h. Lactic acid bacteria were counted on MRS agar medium (CM188, Beijing Luqiao Technology Co., Ltd., Beijing, China), and the culture conditions were 5% CO_2_ and 37 °C for 24 h. The results are presented as the logarithm of the number of bacteria per gram of cecal content (log_10_ CFU/g).

#### DNA extraction and high-throughput sequencing

Bacterial DNA was extracted from ileal digesta with a QIAamp DNA Stool Mini Kit (Qiagen Inc., Valencia, CA, USA) according to the manufacturer’s protocol. The DNA concentrations were measured on a NanoDrop 2000 spectrophotometer (Thermo Scientific, MA, USA). The V3 and V4 regions of the bacterial 16S rRNA gene were amplified with the barcoded primer pair 515F/806R (515F: 5′-GTG CCA GCM GCC GCG GTA A-3′, 806R: 5′-GGA CTA CHV GGG TWT CTA AT-3′) according to previously described methods [24]. After amplification, the PCR products were run on a 2% agarose gel and purified using a QIAquick Gel Extraction Kit (Qiagen, Germany). Pyrosequencing of 16S rDNA was performed on an Illumina HiSeq2500 PE250 platform (Illumina, San Diego, USA) at Novogene Bioinformatics Technology Co., Ltd. (Beijing, China).

#### Sequence processing and bioinformatics analysis

Raw tags were generated by merging paired-end reads using FLASH software (v1.2.7) [25]. High-quality clean tags were obtained by QIIME (v1.7.0) analysis [26], and chimera sequences were removed to obtain effective tags by using the UCHIME algorithm [27]. Sequences were analyzed by UPARSE software (v7.0.1001) and clustered into operational taxonomic units (OTUs) at a similarity level of 97% [28]. Each OTU was annotated with the Greengenes database [29]. Rarefaction curves and Venn diagrams were generated using R software (v2.15.3). Analysis of microbial alpha diversity was conducted using QIIME software [26] with Python scripts. Principal component analysis (PCA) and nonmetric multidimensional scaling (NMDS) were used to evaluate pairwise distances among samples and to establish β-diversity. Linear discriminant analysis (LDA) combined effect size measurements (LEfSe), T tests and Kruskal‒Wallis rank sum tests were used to analyze differences in bacterial abundances among groups. Spearman's correlation analysis was performed to analyze correlations between gut microbiota and other parameters.

### Statistical analysis

SPSS 20.0 software was used to perform the statistical analysis on each group of data. The GLM process was used for statistical analysis. When the interaction was significant, one-way analysis was used, and Duncan’s multiple comparison analysis was used for differences between treatments. *P* < 0.05 was considered to indicate significant differences, and *P* values between 0.05 and 0.10 were considered to indicate trends. In this study, indicators were measured by ELISA in 8 replicates, and the data were acquired from 6 replicates.

## Results

### Effect of MCE on growth performance of broilers with NE

As shown in Table 3, compared with the unchallenged broilers, NE-challenged broilers had significantly reduced d 24 BW, d 30 BW, d 13–24 BWG and d 13–24 FI (*P* < 0.05), significantly increased d 24–30 FCR, d 13–24 FCR and d 13–30 mortality (*P* < 0.05) and nonsignificantly decreased d 24–30 BWG (0.05 < *P* < 0.10). Compared with the control group, dietary MCE supplementation significantly increased d 30 BW and d 24–30 BWG (*P* < 0.05) and significantly decreased d 13–24 FI, d 24–30 FCR and d 13–30 mortality (*P* < 0.05). There was a significant interaction between NE challenge and MCE on mortality during d 13–30 (*P* < 0.05).

**Table 3:** Effects of dietary *Macleaya cordata* extract on growth performance of broiler chickens with necrotic enteritis

NE^1^	MCE^2^, mg/kg	BW, g			BWG, g		FI, g		FCR		d 13–30 mortality rate, %
		d 13	d 24	d 30	d 13–24	d 24–30	d 13–24	d 24–30	d 13–24	d 24–30	
−	0	490	1161^a^	1,565^b^	670^a^	405^b^	1006^a^	743^b^	1.51^b^	1.91^a^	1.92^b^
	350	489	1178^a^	1,644^a^	688^a^	467^b^	978^ab^	783^ab^	1.42^b^	1.70^ab^	0.00^b^
+	0	499	971^b^	1,413^d^	472^b^	442^b^	950^b^	804^a^	2.03^a^	1.88^a^	8.65^a^
	350	495	954^b^	1,492^c^	459^b^	538^a^	939^b^	813^a^	2.05^a^	1.52^b^	1.92^b^
SEM^3^		3.84	7.26	13.7	7.13	13.5	8.38	9.15	0.0252	0.0633	0.608
Main-effect											
NE	−	489	1,162	1,594	673	431	987	769	1.47	1.87	0.64
	+	488	937	1,449	449	508	959	799	2.16	1.62	5.13
MCE	0	495	1,066	1,489	571	423	978	774	1.77	1.89	5.29
	350	492	1,066	1,568	574	503	958	798	1.74	1.61	0.96
*P*-value											
NE		0.207	< 0.001	< 0.001	< 0.001	0.077	0.024	0.037	< 0.001	0.388	0.005
MCE		0.981	0.439	0.003	0.424	0.001	0.049	0.149	0.897	0.008	< 0.001
NE × MCE		0.845	0.237	0.820	0.193	0.386	0.724	0.328	0.174	0.424	0.048

^a-c^Different letters in the shoulder markers in the table indicate significant differences between groups (*P* < 0.05)

Number of replicates for each indicator is 6

^1^NE: Necrotic enteritis

^2^MCE: *Macleaya cordata* extract

^3^SEM: Standard error of mean

### Effect of MCE on intestinal lesion scores of broilers with NE

**Fig. 1:**
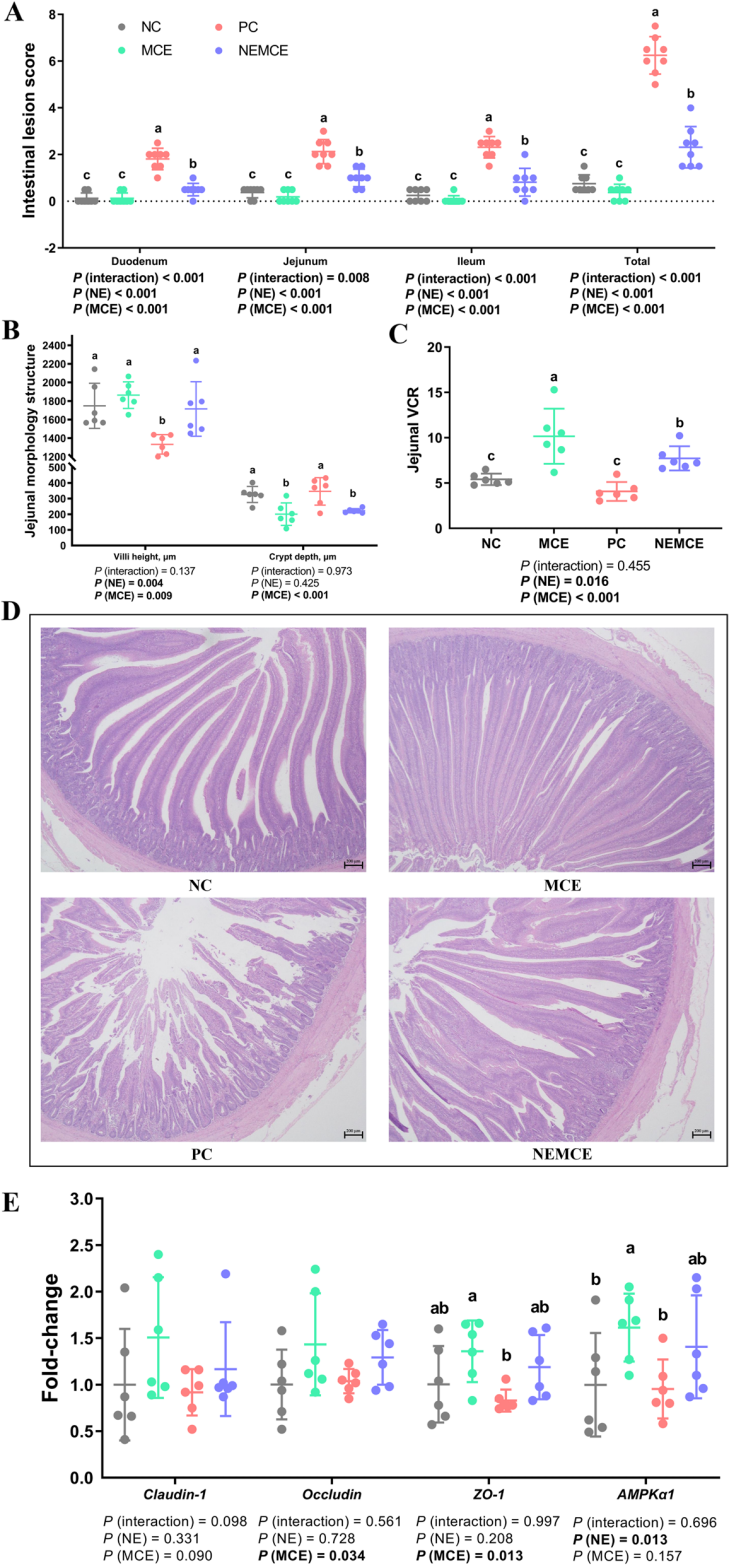
Effects of *Macleaya cordata* extract on the intestinal barrier of broiler chickens with necrotic enteritis. The lesion scores of the duodenum, jejunum, ileum and total intestine (**A**) were analyzed as described in the Materials and methods section. The villus height and crypt depth (**B**) of the jejunum were analyzed by HE staining. The V/C of the jejunum (**C**) was calculated by dividing the villus height by the crypt depth. HE-stained sections were monitored by optical microscopy at 200 × to determine differences in the villus height and crypt depth of the jejunum (**D**). The mRNA levels of *Claudin-1*, *Occludin*, *ZO-1* and *AMPK-α1* in the jejunum (**E**) were analyzed by RT‒PCR. All the data are presented as the mean, with the standard deviation (SD) shown with whiskers. The main effect and interaction effects were analyzed using the general linear model (GLM) procedure, with the *P* values for the main effects written out below each plot. One-way ANOVA and multiple comparisons were performed when interactive effects were significantly different. The lowercase letters on the bar charts indicate significant differences (*P* < 0.05). NC: negative control group; MCE: 350 mg/kg MCE without NE group; PC: positive control group; NEMCE: 350 mg/kg MCE with NE group

As shown in Fig. 1A, compared with the unchallenged broilers, NE-challenged broilers had significantly increased lesion scores in the duodenum, jejunum, ileum, and total intestine on d 30 (*P* < 0.05). Compared with the control group, dietary MCE supplementation significantly reduced the lesion scores of the duodenum, jejunum, ileum, and total intestine (*P* < 0.05). There was a significant interaction between NE challenge and MCE on the lesion scores of the duodenum, jejunum, ileum, and total intestine (*P* < 0.05).

### Effect of MCE on jejunal morphological structure of broilers with NE

As shown in Fig. 1B–D, compared with the unchallenged broilers, NE-challenged broilers had significantly decreased villus height and V/C value in the jejunum on d 30 (*P* < 0.05). Compared with the control group, dietary MCE supplementation significantly increased jejunal villus height and V/C value (*P* < 0.05) and significantly decreased crypt depth (*P* < 0.05).

### Effect of MCE on expression of intestinal barrier-related genes in jejunum of broilers with NE

As shown in Fig. 1E, compared with the unchallenged broilers, NE-challenged broilers had significantly downregulated mRNA levels of *AMPK-α1* in the jejunum on d 30 (*P* < 0.05). Compared with the control group, dietary MCE supplementation significantly upregulated the mRNA levels of *occludin* and *ZO-1* (*P* < 0.05), and there was a trend of dietary MCE supplementation upregulating the mRNA levels of *claudin-1* (0.05 < *P* < 0.10).

### Effect of MCE on cytokine and immunoglobulin levels in the serum of broilers with NE

**Fig. 2:**
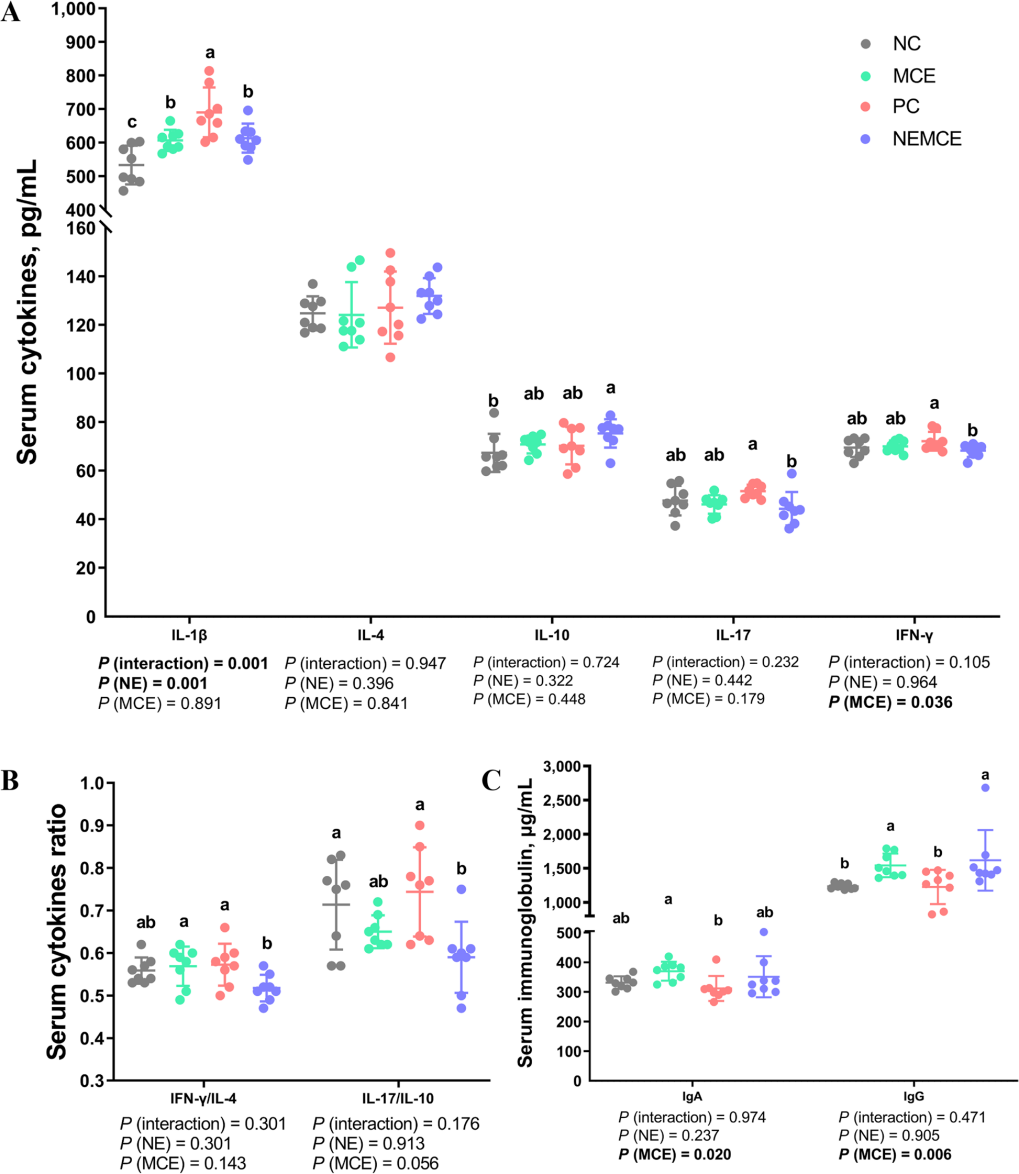
Effects of *Macleaya cordata* extract on the levels of cytokines and immunoglobulins in the serum of broiler chickens with necrotic enteritis. The levels of IL-1β, IL-4, IL-10, IL-17 and IFN-γ (**A**) in the serum were analyzed by ELISA kits. The IFN-γ/IL-4 ratio in serum (**B**) was calculated by dividing the level of IFN-γ by the level of IL-4. The IL-17/IL-10 ratio in serum (**B**) was calculated by dividing the level of IL-17 by the level of IL-10. The levels of IgA and IgG (**C**) in the serum were analyzed by ELISA kits. All the data are presented as the mean, with the standard deviation (SD) shown with whiskers. The main effect and interaction effects were analyzed using the general linear model (GLM) procedure, with the *P* values for the main effects written out below each plot. One-way ANOVA and multiple comparisons were performed when interactive effects were significantly different. The lowercase letters on the bar charts indicate significant differences (*P* < 0.05). NC: negative control group; MCE: 350 mg/kg MCE without NE group; PC: positive control group; NEMCE: 350 mg/kg MCE with NE group

As shown in Fig. 2, compared with the unchallenged broilers, NE-challenged broilers had significantly increased serum concentrations of IL-1β on d 30 (*P* < 0.05). Compared with the control group, dietary MCE supplementation significantly decreased the concentration of IFN-γ (*P* < 0.05), significantly increased the levels of IgA and IgG (*P* < 0.05) and caused a decreasing trend in IL-17/IL-10 (0.05 < *P* < 0.10). There was a significant interaction between NE challenge and MCE on the serum concentration of IL-1β (*P* < 0.05).

### Effect of MCE on cytokine and immunoglobulin levels in jejunal mucosa of broilers with NE

**Fig. 3:**
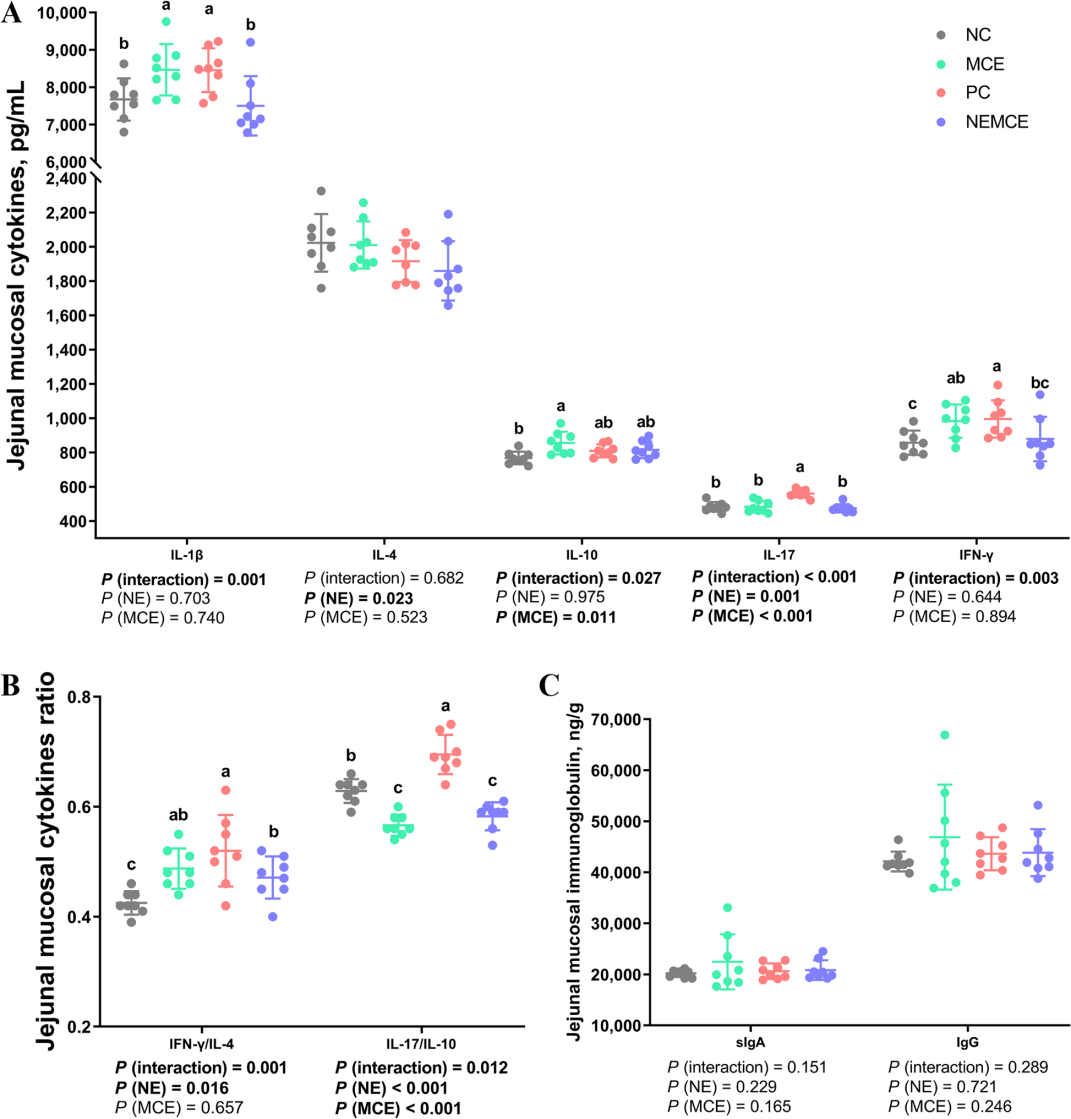
Effects of *Macleaya cordata* extract on the levels of cytokines and immunoglobulins in the jejunal mucosa of broiler chickens with necrotic enteritis. The levels of IL-1β, IL-4, IL-10, IL-17 and IFN-γ (**A**) in the jejunal mucosa were analyzed by ELISA kits. The IFN-γ/IL-4 ratio of the jejunal mucosa (**B**) was calculated by dividing the level of IFN-γ by the level of IL-4. The IL-17/IL-10 ratio of the jejunal mucosa (**B**) was calculated by dividing the level of IL-17 by the level of IL-10. The levels of sIgA and IgG (**C**) in the jejunal mucosa were analyzed by ELISA kits. All the data are presented as the mean, with the standard deviation (SD) shown with whiskers. The main effect and interaction effects were analyzed using the general linear model (GLM) procedure, with the *P* values for the main effects written out below each plot. One-way ANOVA and multiple comparisons were performed when interactive effects were significantly different. The lowercase letters on the bar charts indicate significant differences (*P* < 0.05). NC: negative control group; MCE: 350 mg/kg MCE without NE group; PC: positive control group; NEMCE: 350 mg/kg MCE with NE group

As shown in Fig. 3, compared with the unchallenged broilers, NE-challenged broilers had significantly increased IL-17 concentrations and IFN-γ/IL-4 and IL-17/IL-10 ratios (*P* < 0.05) and significantly decreased IL-4 concentrations in the jejunal mucosa on d 30 (*P* < 0.05). Compared with the control group, dietary MCE supplementation significantly increased the levels of IL-10 (*P* < 0.05) and significantly decreased the concentrations of IL-17 and the ratio of IL-17/IL-10 (*P* < 0.05). There was a significant interaction between NE challenge and MCE on the concentrations of IL-1β, IL-10, IL-17, IFN-γ and the ratios of IFN-γ/IL-4 and IL-17/IL-10 in the jejunal mucosa (*P* < 0.05).

### Effect of MCE on expression of immune-related genes in the jejunum of broilers with NE

**Fig. 4:**
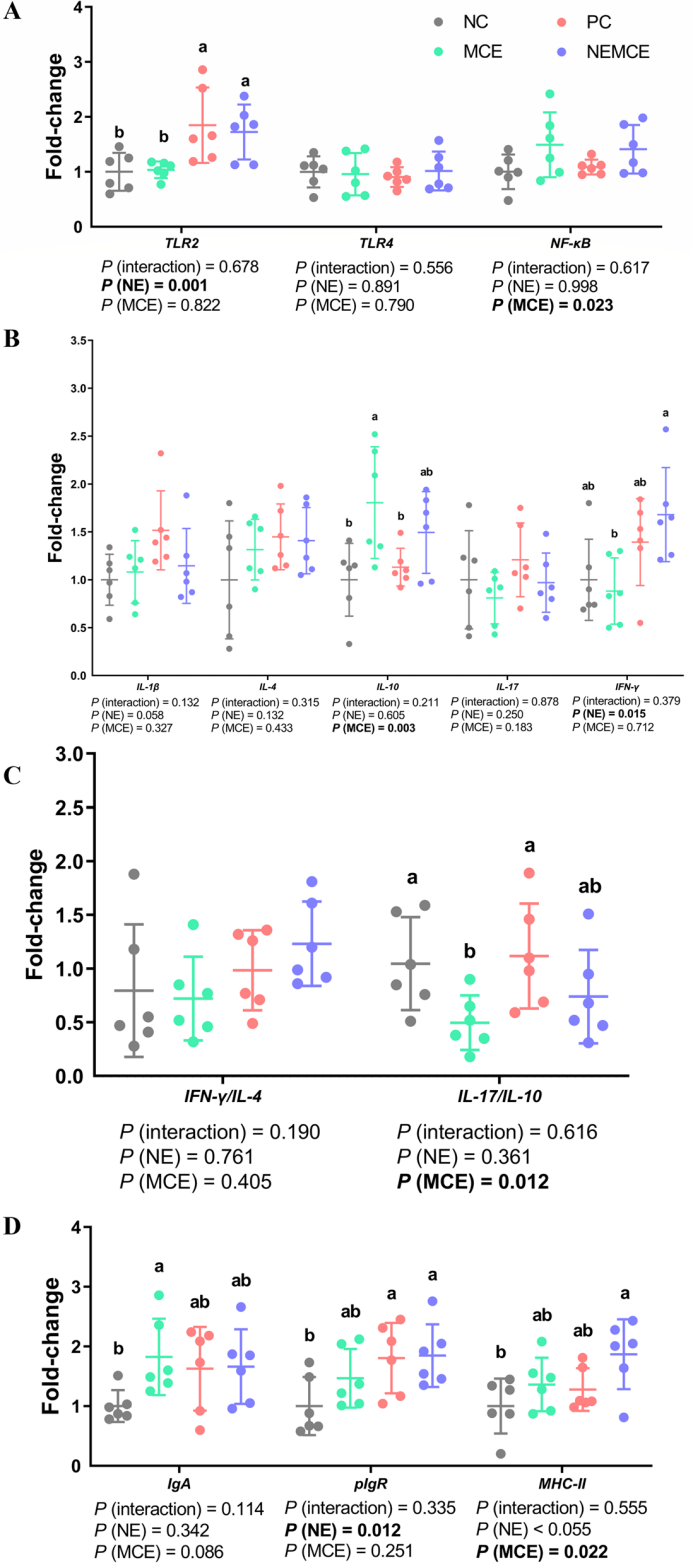
Effects of *Macleaya cordata* extract on the mRNA levels of immune-related genes in the jejunum of broiler chickens with necrotic enteritis. The mRNA levels of *TLR2*, *TLR4* and *NF-κB* (**A**) in the jejunum were analyzed by RT‒PCR. The mRNA levels of *IL-1β*, *IL-4*, *IL-10*, *IL-17* and *IFN-γ* (**B**) in the jejunum were analyzed by RT‒PCR. The *IFN-γ*/*IL-4* ratio in the jejunum (**C**) was calculated by dividing the mRNA level of *IFN-γ* by the mRNA level of *IL-4*. The *IL-17/IL-10* ratio in the jejunum (**C**) was calculated by dividing the mRNA level of *IL-17* by the mRNA level of *IL-10*. The mRNA levels of *IgA*, *pIgR* and *MHC-II* (**D**) in the jejunum were analyzed by RT‒PCR. All the data are presented as the mean, with the standard deviation (SD) shown with whiskers. The main effect and interaction effects were analyzed using the general linear model (GLM) procedure, with the *P* values for the main effects written out below each plot. One-way ANOVA and multiple comparisons were performed when interactive effects were significantly different. The lowercase letters on the bar charts indicate significant differences (*P* < 0.05). NC: negative control group; MCE: 350 mg/kg MCE without NE group; PC: positive control group; NEMCE: 350 mg/kg MCE with NE group

As shown in Fig. 4, compared with the unchallenged broilers, NE-challenged broilers had significantly upregulated mRNA levels of *TLR2*, *IFN-γ* and *pIgR* in the jejunum on d 30 (*P* < 0.05), and there was a trend of upregulated mRNA levels of *IL-1β* and *MHC-II* (0.05 < *P* < 0.10). Compared with the control group, dietary MCE supplementation significantly upregulated the mRNA levels of *NF-κB*, *IL-10* and *MHC-II* in the jejunum on d 30 (*P* < 0.05), significantly downregulated the mRNA levels of *IL-17/IL-10* (*P* < 0.05) and caused a trend of *IgA* mRNA upregulation (0.05 < *P* < 0.10).

### Effect of MCE on cecal microbiota of broilers with NE

#### Concentrations of *Clostridium perfringens *and lactic acid bacteria

**Fig. 5:**
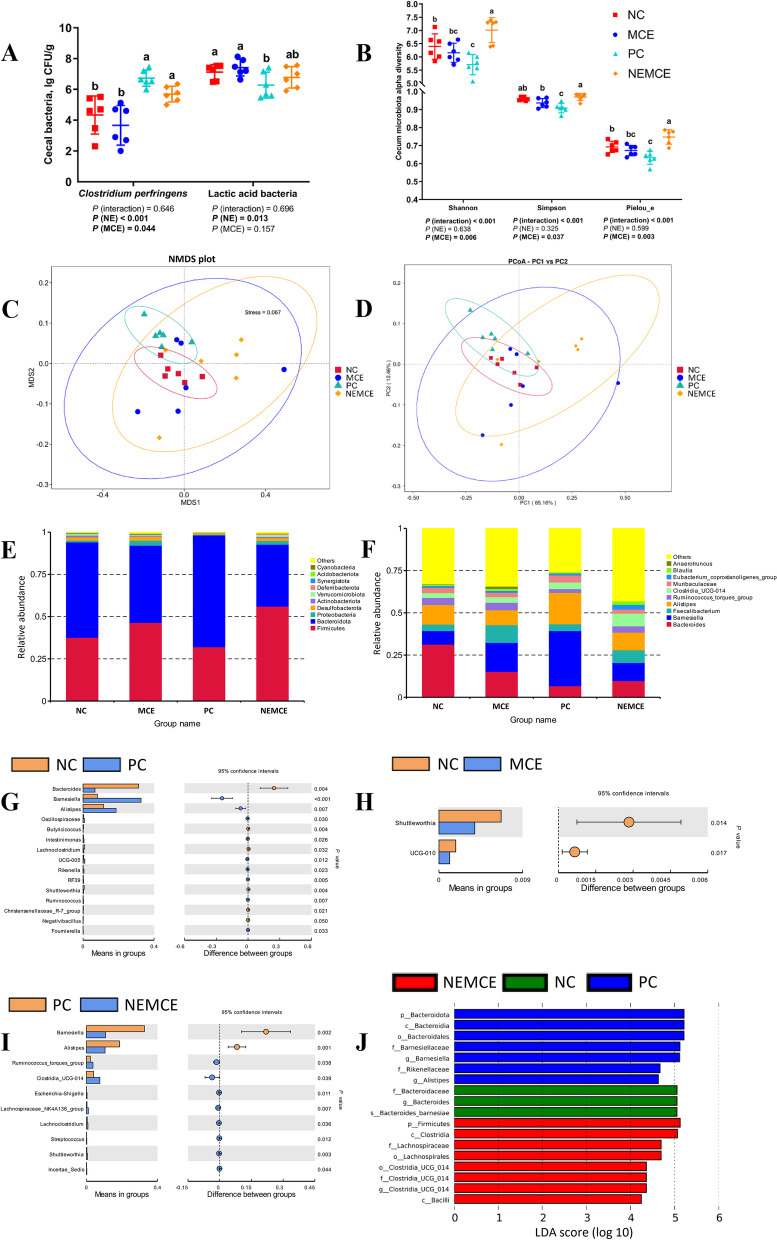
Effects of *Macleaya cordata* extract on the cecal microbiota of broiler chickens with necrotic enteritis. The abundances of *Clostridium perfringens* and lactic acid bacteria (**A**) in the cecum were determined by the culture count method. The alpha diversity of the cecal microbiota of broiler chickens was analyzed by the Shannon index, Simpson index and Pielou_e (**B**). The beta diversity of the cecum microbiota of broiler chickens was analyzed by NMDS (**C**) and PCA (**D**). Top 10 microbes in the cecum of broiler chickens on d 30 at the phylum level (**E**) and genus level (**F**). Differential microbes in the cecum of broiler chickens according to T test (**G–I**). Differential microbes in the cecum of broiler chickens according to LEfSe analysis (LDA score is greater than 4) (**J**). NC: negative control group; MCE: 350 mg/kg MCE without NE group; PC: positive control group; NEMCE: 350 mg/kg MCE with NE group

As shown in Fig. 5A, compared with the unchallenged broilers, NE-challenged broilers had significantly increased abundances of *Clostridium perfringens* in the cecum on d 30 (*P* < 0.05) and significantly decreased abundances of lactic acid bacteria. Compared with the control group, dietary MCE supplementation significantly reduced the abundance of *Clostridium perfringens* (*P* < 0.05).

#### Alpha diversity

As shown in Fig. 5B, NE challenge had no significant effect on the α diversity of the microbiota in the cecum (*P* > 0.05). Compared with the control group, dietary MCE supplementation significantly increased the Shannon, Simpson and Pielou_e indices (*P* < 0.05). There was a significant interaction between NE challenge and MCE on the Shannon, Simpson and Pielou_e indices (*P* < 0.05).

#### Beta diversity

As seen from the NMDS (Fig. 5C) and PCA (Fig. 5D) plots, the NC and PC groups could be better separated, demonstrating that NE challenge exerted a significant effect on the community composition of the cecum microbiota. Compared to the control group, the distance between sample points was greater in the MCE and NEMCE groups, demonstrating that dietary MCE supplementation had a more significant effect on disrupting the community composition of the microbiota in the cecum of broiler chickens.

#### Top 10 microbes at the phylum and genus levels in the cecum

As shown in Fig. 5E and Table S1, at the phylum level, compared with the unchallenged broilers, there was a trend toward a reduction in the proportions of Desulfobacterota and Synergistota in the cecum of the NE-challenged broilers (0.05 < *P* < 0.10). Compared with the control group, dietary MCE supplementation significantly increased the proportions of Firmicutes and Proteobacteria (*P* < 0.05) and significantly decreased the proportion of Bacteroidetes (*P* < 0.05) in the cecum on d 30.

As shown in Fig. 5F and Table S2, at the genus level, compared with the unchallenged broilers, NE-challenged broilers had significantly increased proportions of *Clostridia_UCG-014* and *Eubacterium_coprostanoligenes_group* in the cecum (*P* < 0.05), significantly decreased proportions of *Bacteroides* and *Anaerotruncus* (*P* < 0.05) and a trend toward higher proportions of *Barnesiella* and *Alistipes* (0.05 < *P* < 0.10). Dietary MCE supplementation significantly increased the proportion of *Faecalibacterium* in the cecum on d 30 (*P* < 0.05), significantly decreased the proportion of *Alistipes* (*P* < 0.05) and tended to increase the proportion of *Blautia* (0.05 < *P* < 0.10).

#### T test to analyze differences in microbe abundance

As shown in Fig. 5G, compared with the unchallenged broilers, NE-challenged broilers had significantly increased relative abundances (*P* < 0.05) and significantly decreased relative abundances of *Bacteroides*, *Butyricicoccus*, *Lachnoclostridium*, *Shuttleworthia*, *Christensenellaceae_R-7_group* and *Negativibacillus* (*P* < 0.05).

As shown in Fig. 5H, compared with the control group, dietary MCE supplementation significantly reduced the relative abundances of *Shuttleworthia* and *UCG-010* (*P* < 0.05).

As shown in Fig. 5I, compared with the control group, dietary MCE supplementation significantly increased the relative abundances of *Ruminococcus_torques_group*, *Clostridia_UCG-014*, *Escherichia-Shigella*, *Lachnospiraceae_NK4A136_group*, *Lachnoclostridium*, *Streptococcus*, *Shuttleworthia* and *Incertae_Sedis* in the cecum on d 30 (*P* < 0.05) and significantly decreased the relative abundances of *Barnesiella* and *Alistipes* (*P* < 0.05).

#### LEfSe analysis of cecal microbiota

As shown in Fig. 5J, compared with the unchallenged broilers, NE-challenged broilers had significantly increased relative abundances of Bacteroidota, Bacteroidia, Bacteroidales, Barnesiellaceae, *Barnesiella*, Rikenellaceae and *Alistipes* in the cecum (*P* < 0.05). Compared with the control group, dietary MCE supplementation significantly (*P* < 0.05) increased the relative abundances of Firmicutes, Clostridia, Lachnospiraceae, *Clostridia_UCG_014* and Bacilli in the cecum on d 30.

#### Correlation heatmap of differential parameters and cecal microbes

**Fig. 6:**
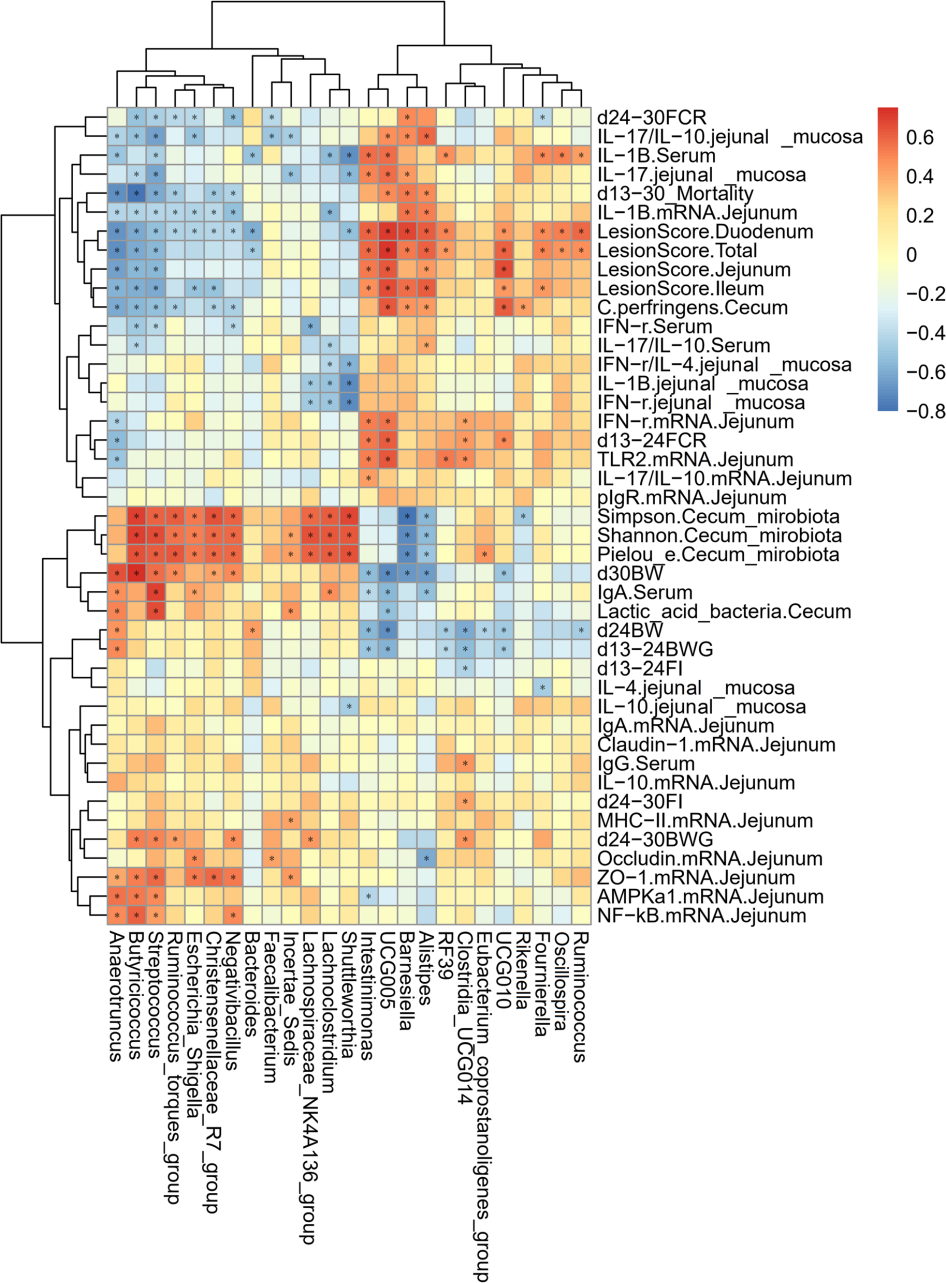
Correlation heatmap of differential microbes in the cecum and differential parameters of broiler chickens. Spearman's correlations were calculated for all significantly different parameters and differential cecal microbes at the genus level. Colors of squares represent *r* values of Spearman's correlation coefficient. ^*^P < 0.05

To explore the correlation between the gut microbiota of broiler chickens and the growth performance and gut health of broiler chickens, we performed a Spearman correlation analysis based on the above data of different cecal microbes at the genus level and different parameters (Fig. 6) to identify microbes that are associated with gut health. As shown in Fig. 6, growth performance (BW, BWG, FI, FCR and mortality) showed a significant positive correlation with *Anaerotruncus*, *Butyricoccus*, *Streptococcus*, *Ruminococcus_torques_group*, *Escherichia_Shigella*, *Christensenellaceae_R7_group, Negativibacillus*, *Bacteroides* and *Faecalibacterium* and a significant negative correlation with *Intestinimonas*, *UCG-005*, *Barnesiella*, *Alistipes*, *RF39*, *Clostridia_UCG014*, *Eubacterium_coprostanoligenes_group*, *UCG-010* and *Ruminococcus*. Intestinal lesion scores had a significant positive correlation with *Intestinimonas*, *UCG-005*, *Barnesiella*, *Alistipes*, *RF39*, *UCG-010*, *Fournierella*, *Oscillospira* and *Ruminococcus* and a significant negative correlation with *Anaerotruncus*, *Butyricoccus*, *Streptococcus*, *Ruminococcus_torques_group*, *Escherichia_Shigella*, *Christensenellaceae_R7_group, Negativibacillus*, *Bacteroides* and *Shuttleworthia*. The expression of tight junction proteins and *AMPK-α1* genes in the jejunum showed a significant positive correlation with *Anaerotruncus*, *Butyricoccus*, *Streptococcus*, *Escherichia_Shigella*, *Christensenellaceae_R7_group, Negativibacillus*, *Faecalibacterium* and *Incertae_Sedis* and a significant negative correlation with *Intestinimonas* and *Alistipes*. The levels of pro-inflammatory cytokines in the serum showed a significant positive correlation with *Intestinimonas*, *UCG-005*, *Alistipes*, *RF39*, *Fournierella*, *Oscillospira* and *Ruminococcus* and a significant negative correlation with *Anaerotruncus*, *Butyricoccus*, *Streptococcus*, *Negativibacillus*, *Bacteroides*, *Lachnospiraceae_NK4A136_group*, *Lachnoclostridium* and *Shuttleworthia*. The serum levels of immunoglobulins showed a significant positive correlation with *Anaerotruncus*, *Streptococcus*, *Escherichia_Shigella*, *Lachnoclostridium* and *Clostridia_UCG014* and a significant negative correlation with *Intestinimonas*, *UCG-005* and *Alistipes*. The levels of pro-inflammatory cytokines in the jejunal mucosa showed a significant positive correlation with *Intestinimonas*, *UCG-005*, *Barnesiella* and *Alistipes* and a significant negative correlation with *Anaerotruncus*, *Butyricoccus*, *Streptococcus*, *Escherichia_Shigella*, *Faecalibacterium*, *Incertae_Sedis*, *Lachnospiraceae_NK4A136_group*, *Lachnoclostridium* and *Shuttleworthia*. The levels of anti-inflammatory cytokines in the jejunal mucosa showed a significant negative correlation with *Shuttleworthia* and *Fournierella*. The expression of immune-related genes in the jejunum showed a significant positive correlation with *Intestinimonas*, *UCG-005*, *Barnesiella*, *Alistipes*, *RF39*, *Clostridia_UCG014*, and *Incertae_Sedis* and a significant negative correlation with *Anaerotruncus*, *Butyricoccus*, *Streptococcus*, *Ruminococcus_torques_group*, *Escherichia_Shigella*, *Christensenellaceae_R7_group, Negativibacillus* and *Lachnoclostridium*. The abundance of *Clostridium perfringens* in the cecum showed a significant positive correlation with *UCG-005*, *Barnesiella*, *Alistipes*, *UCG-010* and *Rikenella* and a significant negative correlation with *Anaerotruncus*, *Butyricoccus*, *Streptococcus*, *Ruminococcus_torques_group*, *Christensenellaceae_R7_group* and *Negativibacillus.* The abundance of lactic acid bacteria in the cecum showed a significant positive correlation with *Anaerotruncus*, *Streptococcus* and *Incertae_Sedis* and a significant negative correlation with *UCG005*. The α diversity of the cecal microbiota showed a significant positive correlation with *Butyricoccus*, *Streptococcus*, *Ruminococcus_torques_group*, *Escherichia_Shigella*, *Christensenellaceae_R7_group, Negativibacillus*, *Incertae_Sedis*, *Lachnospiraceae_NK4A136_group*, *Lachnoclostridium*, *Shuttleworthia* and *Eubacterium_coprostanoligenes_group* and a significant negative correlation with *Barnesiella*, *Alistipes* and *Rikenella*. Therefore, based on the results of the Spearman correlation analysis, we can conclude that the cecum microbes that were associated with beneficial growth performance were *Anaerotruncus*, *Butyricoccus*, *Streptococcus*, *Ruminococcus_torques_group*, *Escherichia_Shigella*, *Christensenellaceae_R7_group, Negativibacillus*, *Bacteroides* and *Faecalibacterium*. The microbes that were associated with anti-inflammatory properties were *Anaerotruncus*, *Butyricoccus*, *Streptococcus*, *Ruminococcus_torques_group*, *Escherichia_Shigella*, *Christensenellaceae_R7_group, Negativibacillus*, *Bacteroides*, *Faecalibacterium*, *Incertae_Sedis*, *Lachnospiraceae_NK4A136_group*, *Lachnoclostridium* and *Shuttleworthia*. The microbes that were associated with impaired growth performance were *Intestinimonas*, *UCG-005*, *Barnesiella*, *Alistipes*, *RF39*, *Clostridia_UCG014*, *Eubacterium_coprostanoligenes_group*, *UCG-010* and *Ruminococcus*. The microbes that were associated with inflammation in this study were *Intestinimonas*, *UCG-005*, *Barnesiella*, *Alistipes*, *RF39*, *Clostridia_UCG014*, *Fournierella*, *Oscillospira* and *Ruminococcus*.

## Discussion

### Effect of MCE on growth performance of broilers with NE

NE is an intestinal toxemic disease that is caused by *Clostridium perfringens* and commonly occurs in broilers between 2 and 5 weeks of age, with mortality rates ranging from 2% to 10% and up to 50%. Geier et al. [30] showed that NE can increase broiler carcass ratios, mortality rates and intestinal lesion numbers and decrease BWG. The main cause of reduced growth performance is the coccidia-induced leakage of proteins (including plasma) into the intestinal lumen, which promotes the rapid multiplication of toxin-producing *Clostridium perfringens* and its pathogenicity and decreases the digestive and absorptive capacity of the small intestine [6]. Consistent with previous studies [22, 31], this study observed a significant negative effect of NE on BW and BWG in broilers.

In our study, MCE was effective in ameliorating the NE-induced reduction in growth performance. The main active ingredient in *Macleaya cordata* is sanguinarine, which has been used as a feed additive for poultry [32]. Previous studies found that the addition of 150 mg/kg MEC to the diet of broilers with NE controlled the negative effects of NE on the growth performance of broilers [10]. The administration of 200 mg/L MCE via drinking water significantly increased the BW and reduced the proportion of abdominal fat in broilers [33]. Dietary supplementation with 120 mg/kg MCE significantly increased growth performance and decreased intestinal lesion scores of broilers with NE [15]. Jeroch et al. [34] reported that the addition of MCE to wheat diets significantly improved the growth performance of turkeys. Vieira et al. [32] also found that dietary supplementation with 25 and 50 mg/kg MCE significantly increased the mean BW of broilers on d 21. It was reported that dietary supplementation with 0.6 mg/kg MCE (0.4 mg/kg protopine and 0.2 mg/kg allotypotopine) significantly increased average daily BWG and significantly decreased the FCR of broilers during d 0–42 [12]. It has been found that dietary supplementation with 15, 50 and 150 mg/kg MCE significantly increased the FI of yellow-feathered broilers on d 56, and dietary supplementation with 50 mg/kg MCE significantly reduced the FCR of white-feathered broilers during d 1–42 [19].

The results of the present study are like those of previous studies. In the present study, we found that dietary supplementation with 350 mg/kg MCE significantly increased the mean BW and BWG and significantly reduced FI, FCR and mortality in broilers. In this study, we found that MCE improved the growth performance of chickens with NE, which may be related to the beneficial effect of MCE in protecting the intestinal barrier. Therefore, in the next step, we evaluated the effects of MCE on indicators related to the intestinal barrier of broilers.

### Effect of MCE on intestinal barrier function of broilers with NE

A healthy intestinal system is extremely important for the growth performance of broiler chickens. A healthy intestinal system requires a normal physiological state with normal nutrient digestion and absorption, immune function, and gut microbiota. The gut is the first target organ for pathogenic bacteria and dietary MCE supplementation, so attention must be paid to intestinal inflammation. NE causes intestinal inflammation in broilers, disrupting the function of the intestinal barrier and increasing intestinal permeability [35]. In farm production, NE in broiler chickens has been found to cause symptoms such as intestinal damage, reduced performance, and increased mortality. Given the characteristics of NE, intestinal health and function are the focus of this study. The intestinal lesion score, intestinal tissue morphology structure and expression of tight junction protein genes are important indicators for assessing intestinal health and disease recovery [36]. Occludin and claudins constitute the major components of intestinal tight junctions and regulate the barrier properties of the paracellular space by forming tight junction chains. ZO-1 belongs to the zonula occludin family of proteins. ZO family proteins play key roles in the assembly of intestinal tight junctions by acting as cytoplasmic scaffolding proteins that link transmembrane proteins to the intracellular actin cytoskeleton [37]. AMP-activated protein kinase (AMPK) is a stress-activated kinase that regulates the ATP:AMP ratio, which is an intracellular energy sensor. A previous study demonstrated that Picroside III attenuated DSS-induced colitis by promoting colonic mucosal wound healing and epithelial barrier function recovery via the activation of AMPK [38].

In this study, we found that NE significantly increased intestinal lesion scores, decreased jejunal villus height and V/C and downregulated the mRNA expression of *AMPK-α1* in the jejunum. These findings indicated that the NE model had been successfully established. Broilers with NE had severe intestinal damage, and the morphological structure of the intestine had been destroyed. This is relatively like previous findings that NE in broiler chickens significantly increases the lesion scores of the intestinal tract, such as the duodenum and jejunum [36, 39]. Necrotic enterocolitis lesions are among the most severe of all diseases that affect the chicken intestine [40]. This is probably the main cause of the reduced growth performance of broilers caused by NE.

Our study found that dietary MCE supplementation significantly increased jejunal villus height, V/C, and the mRNA expression of jejunal *occludin* and *ZO-1* and reduced intestinal lesion scores and jejunal crypt depth. This suggested that MCE alleviated the intestinal damage and improved the intestinal barrier function of broilers with NE. Previous studies have shown that dietary supplementation with 100 mg/kg MCE significantly improved growth performance, reduced serum levels of lipid peroxide, corticosterone, uric acid and FITC-D, and reduced the mRNA levels of *IL-6*, *TNF-α* and *iNOS* in the ileum of heat-stressed broilers [13]. Dietary supplementation with 120 mg/kg MCE significantly reduced growth performance and decreased intestinal lesion scores of broilers with NE [15]. Dietary supplementation with 150 mg/kg MCE significantly increased the FI, BWG and breast meat percentage and decreased the lesion scores of the duodenum, jejunum and ileum in broilers with NE [10]. Sanguinarine is the major alkaloid (isoquinoline alkaloid) of MCE [41]. When administered at 1 mg/kg by gavage, sanguinarine alleviated indomethacin-induced intestinal inflammation in rats and significantly reduced the colonic mucosal damage index score and tissue damage index, probably because sanguinarine promoted ZO-1 expression, inhibited TNF-α, IL-1β and IL-6 expression, suppressed inflammation, and protected intestinal barrier function, thus alleviating indomethacin-induced intestinal damage in rats [42]. Dietary supplementation with 0.7 mg/kg sanguinarine significantly improved growth performance, reduced serum glucose, uric acid and urea nitrogen levels and increased the ratio of villus height to crypt depth in the jejunum and ileum of yellow-feathered broilers [43]. Dietary sanguinarine supplementation significantly increased BWG, reduced the crypt depth in the ileum, and improved the intestinal morphological structure in broilers that were challenged with *Salmonella* [44]. The results of this study are like those of previous studies, and we hypothesize that there are multiple mechanisms by which MCE alleviates intestinal damage and protects the intestinal barrier of broilers with NE. In addition to the possible bacteriostatic properties of MCE, it may also indirectly suppress the number of pathogenic bacteria in the gut by modulating the immune function of broilers, thereby alleviating pathogen-induced intestinal inflammation and intestinal mucosal damage. Therefore, the effect of MCE on the immune function of broiler chickens with NE was investigated immediately afterward.

### Effect of MCE on immune function of broilers with NE

The gut is not only an organ where digestion and absorption occur but also the largest immune organ in the body. The immune system is the "army" that protects the life of an animal. The immune system plays defensive, monitoring, and self-stabilizing roles against disease threats [45]. Cell surface markers, such as MHC-II and CD80, are considered markers of dendritic cell maturation in animals [46]. Toll-like receptors (TLRs), which are a class of pattern recognition receptors (PRRs) that can be activated by certain endogenous molecules and conserved molecular components of microbes, are widely involved in the recognition of pathogen-associated molecular patterns (PAMPs) that are found on pathogens, initiate natural immune responses and are host defenses. When TLR2 binds to pathogen-associated molecular patterns, it activates specific immune signaling pathways that lead to the activation of NF-κB. As a result, NF-κB dissociates from IκB, translocates to the nucleus, upregulates the transcription levels of related genes, and produces proinflammatory cytokines such as IL-1β, IL-6 and TNF-α, which in turn recruit and activate many immunoreactive cells and enhance the body's immune response [47, 48]. IL-17 is a proinflammatory cytokine that is produced by Th17 cells and mediates the inflammatory response by directly or indirectly inducing the production of a variety of inflammatory factors and chemokines [49]. Th1 cells mainly secrete proinflammatory cytokines, including IL-1β, IFN-γ, TNF-α and inflammatory chemokines, which mediate the various stages of inflammation and immune responses. Th2 cells mainly secrete anti-inflammatory cytokines, including IL-4, IL-10 and TGF-β, which stimulate B-cell differentiation and proliferation, produce immunoglobulins and mediate humoral immune function. sIgA prevents pathogenic bacteria from adsorbing and invading epithelial cells, thereby protecting intestinal epithelial cells from intestinal toxins and pathogenic microorganisms [50]. pIgR, which is a key component of sIgA, transports polymeric IgA from the lamina propria to the luminal mucin and is expressed on the basolateral surface of both the liver and intestinal epithelium [51].

In the present study, we found that NE in broiler chickens significantly increased serum IL-1β levels, jejunal mucosa IL-17, IFN-γ/IL-4 and IL-17/IL-10 levels, and mRNA levels of *TLR2*, *IFN-γ* and *pIgR* in the jejunum and significantly decreased jejunal mucosal IL-4 levels. This indicated that NE increased the levels of pro-inflammatory cytokines in the serum and jejunum, inhibited the levels of anti-inflammatory cytokines, and led to an inflammatory state in the intestines of broilers. Previous studies reported similar findings. NE of broilers was reported to significantly increase Th17/Treg ratios (blood and ileal Th17 cell%, blood Th17/Treg ratios, IL-17 levels and IL-17/IL-10 ratios), NO levels, blood lysozyme activity and IL-1β ratios, intestinal immune cell ratio and activity (ileal Tc%, Treg% and monocyte phagocytic activity), and intestinal inflammatory gene (*TLR2*, *NF-κB*, *TNF-α* and *IL-6*) expression levels [36]. One study found that NE significantly upregulated the mRNA levels of *IL-1β*, *IL-6* and *IL-8* in the intestine of broilers [52]. It was also found that NE significantly upregulated the mRNA levels of *TLR2*, *TLR4* and *IFN-γ* in the jejunum of broilers [53]. Like the present study, previous studies showed that NE increased the levels of inflammatory cytokines and led to intestinal inflammation.

Our study showed that dietary MCE supplementation significantly increased serum IgA and IgG levels, jejunal mucosal IL-10 levels, and jejunal mRNA levels of *NF-κB*, *IL-10* and *MHC-II* and significantly decreased serum IFN-γ levels, jejunal mucosal IL-17 levels, the ratio of IL-17/IL-10, and the jejunal mRNA level of *IL-17/IL-10*. Reportedly, the addition of 1% MCE (blood root base content of 6.5 mg/mL) to drinking water significantly increased serum IgG levels and decreased serum IL-1β levels in broilers [14]. One study found that dietary supplementation with 0.6 mg/kg MCE (0.4 mg/kg protopine and 0.2 mg/kg allotypotopine) in broiler diets significantly downregulated liver *TLR4*, *MyD88*, *NF-κB* and *NLRP3* mRNA levels [12]. It was also found that dietary supplementation with 100, 150 and 200 mg/kg MCE significantly reduced serum IL-6 and TNF-α levels but did not affect serum IgG, IgA and IgM levels in laying hens [54]. However, other studies have found that supplementation of drinking water with 25, 50 and 100 mg/L MCE (containing 11,078 mg/kg sanguinarine and 4,320 mg/kg chelerythrine) significantly downregulated the jejunal mRNA level of *iNOS* in broilers but had no significant effect on the mRNA levels of *IL-1β* and *IL-10* [33]. The results of our study revealed that the levels of pro-inflammatory cytokines were decreased, and the levels of anti-inflammatory cytokines were increased, alleviating intestinal inflammation. Furthermore, MCE increased the levels of immunoglobulins, thus enhancing the immune function of broilers, indirectly inhibiting pathogenic bacteria in the intestine, reducing pathogenic bacterial metabolite production and damage to intestinal epithelial cells, and ultimately protecting the intestinal mucosa. In addition, MCE may exert a direct bacteriostatic effect. Many microbes reside in the gut, especially in the cecum. Mucosal immune function and status can also interact with the gut microbiota. A healthy and normal gut microbiota is also essential for the recovery of intestinal health in broilers with NE. Therefore, as a next step, we explored the effect of MCE on the gut microbiota of broilers with NE.

### Effect of MCE on gut microbiota of broilers with NE

The gut microbiota is an important organ that influences nutrient absorption, feed digestibility, energy utilization and productivity in poultry [55]. The gut microbiota is the largest symbiotic ecosystem with the host and has been shown to play an important role in maintaining gut homeostasis. A healthy gut microbiota can maintain a good and mutually beneficial symbiotic relationship with the animal. The composition of the gut microbiota is controlled by the normal mucosal immune system, and a symbiotic microbiome regulates the maturation of the mucosal immune system. In contrast, a pathogenic microbiome causes immune dysfunction and affects the balance of the gut microbiome, leading to the development of intestinal diseases [17]. Early colonization and establishment of the gut microbiota in chicks can alter gut morphology and physiology as well as susceptibility to infectious diseases [56].

Previous studies on the effect of NE on the gut microbiota of broiler chickens have shown that NE significantly increased the relative abundances of *Staphylococcus* and *Turicibacter* in the ileum and significantly decreased the alpha diversity of the ileal microbiota [36]. One study found that NE significantly increased the relative abundances of *Clostridium *sensu stricto 1 and *Escherichia_Shigella* in the jejunum of broiler chickens and significantly decreased the relative abundances of *Lactobacillus*, *Bifidobacterium* and *Phaseolus_acutifolius_ tepary_bean* as well as the alpha diversity of the jejunal microbiota [57]. It was also found that NE significantly reduced the number of unique OTUs in the ileum and cecum of broiler chickens, significantly increased the abundances of *Romboutsia*, *Staphylococcus*, *Bacteroides*, *Weissella*, and *Faecalibacterium* in the ileum and *Alistipes, Megamonas,* and *Phascolarctobacter* in the cecum, and significantly decreased the relative abundances of *Bacteroides*, *Lactobacillus* and *Tyzzerella* in the cecum [58]. In the present study, NE significantly increased the abundance of *Clostridium perfringens* and the relative abundances of *Alistipes*, *Barnesiella*, *Intestinimonas*, *UCG-005*, *RF39* and *Oscillospiraceae* in the cecum and significantly decreased the abundance of lactic acid bacteria and the relative abundances of *Anaerotruncus*, *Butyricicoccus* and *Bacteroides*.

Both *Alistipes* and *Bacteroides* are important bacteria that produce short-chain fatty acids (SCFAs) [59, 60]. SCFAs play an important role in intestinal physiology [61]. SCFAs, especially butyrate, can serve as an energy source for colonic epithelial cells [62]. In addition, SCFAs negatively affect the expression of virulence factors of bacterial pathogens [63]. In the present study, NE reduced the abundance of the first major genus *Bacteroides* in the cecum of normal broiler chickens, allow the abundance of *Alistipes*, which also function as SCFA producers, to increase. A significant increase in *Alistipes* with pro-inflammatory properties has also been reported in obese populations [64]. Previous work has shown that *Barnesiella* is an important member of the flora in patients with depression [65]. Specialized anaerobic bacteria belonging to the genus *Barnesiella* have been reported to reduce the colonization of the intestinal tract by vancomycin-resistant *Enterococcus faecalis* in mice, suggesting a greater ability to occupy ecological niches in an anaerobic environment [66]. In the present study, the higher production of CO_2_ by proliferating *Clostridium perfringens* generated an anaerobic environment that was more favorable for the survival of *Barnesiella*. Previous studies have reported that *Clostridium perfringens* is significantly enriched in high-fat diet-fed mice and positively correlates with obesity [67] and that *Terminalia bellirica* ethanol extract can improve nonalcoholic fatty liver disease in mice by reducing the abundance of *Intestinimonas* [68]. It has also been reported that *Intestinimonas* produces butyric acid to maintain intestinal integrity and enhance the barrier function of damaged intestines [69]. The enrichment of *Intestinimonas* in broilers with NE may also be a protective mechanism associated with disease recovery and not part of the pathogenic mechanism that induces or maintains the disease. A positive correlation between RF39 and increased inflammatory cytokines has been reported [70]. RF39 is associated with increased adiposity in rats fed a Western high-fat diet [71]. *RF39* is significantly increased in the intestine of adult offspring of maternal mice that were fed a high-fat diet and is associated with steatohepatitis [72]. The relative abundance of *RF39* was significantly increased in the intestines of rats with myocardial ischemia‒reperfusion injury and significantly decreased after electroacupuncture treatment [73]. Both *UCG-005* and *UCG-010* in this study belong to the family Oscillospiraceae, and Oscillospiraceae have been reported to be key pathogenic intestinal bacteria that are related to inflammation in the development of colitis [74]. *Anaerotruncus* in the gut has been shown to promote fiber digestion and the production of SCFAs, which may allow for improvements in intestinal immune function and morphological structures in animals [60, 75]. In previous studies, members of the genus *Butyricicoccus* were identified as produces of butyrate in the gut and showed good anti-inflammatory effects in human and animal experiments [76]. *Bacteroides* is one of the most abundant genera in the cecum of chickens [77]. *Bacteroides* has been reported to be associated with propionic acid production levels in the chicken gut [78]. The present study shows that NE significantly increased the abundance of bacteria (*Alistipes*, *Barnesiella*, *Intestinimonas*, *UCG-005*, *RF39* and *Oscillospiraceae,* among others) that are associated with inflammation, obesity and depressive disorders in the cecum of broiler chickens and significantly decreased the abundance of SCFA-producing bacteria (*Anaerotruncus*, *Butyricicoccus* and *Bacteroides*).

Previous studies on the effects of dietary MCE supplementation on the gut microbiota showed that dietary MCE mainly altered the microbiota in the anterior half of the chicken gut, promoting the proliferation of *Lactobacillus*, inhibiting the colonization of *Escherichia coli*, and upregulating amino acid, vitamin and secondary bile acid biosynthetic pathways while avoiding the accumulation of antibiotic resistance genes [19]. In one study, dietary supplementation with 100 mg/kg MCE significantly increased the diversity of microbiota in the ileum of Snowy Peak blackbone chickens, and dietary supplementation with 200 mg/kg MCE significantly increased the relative abundances of *Lactobacillus* and *Aeriscardovia* in the ileum and significantly increased the abundance of *Bacteroidetes* and *Deferribacteres* in the cecum. The relative abundance of Firmicutes in the cecum was significantly decreased [20]. It was also found that dietary supplementation with 50 mg/kg MCE (1.5% hematoxylin) significantly increased the abundance of *Lactobacillus* and the concentrations of acetate, propionate, butyrate and total SCFAs in the ileum and cecum of weaned piglets and significantly decreased the abundance of *Escherichia coli* and *Salmonella *spp. in the ileum and cecum and the concentrations of ammonia [79]. It was also found that instillation of 5 mg/kg/d MCE in heat-stressed mice significantly increased the relative abundances of *Lactobacillus*, *Desulfovibrio* and *Helicobacter* in the cecum and significantly decreased the relative abundances of *Muribaculaceae*, *Bacteroides* and *Lachnospiraceae NK4A136* [80].

Previous findings are like our present study, which showed that MCE increased the alpha diversity of the cecal microbiota. Reportedly, a higher diversity of the gut microbiota is beneficial for its stability and for gut health, and it reduces susceptibility to foreign pathogens [81]. In our present study, MCE significantly increased the relative abundances of *Streptococcus*, *Ruminococcus_torques_group* and *Lachnospiraceae_NK4A136_group* in the cecum and significantly decreased the abundance of *Clostridium perfringens* and the relative abundances of *UCG-010*, *Barnesiella* and *Alistipes*. *Streptococcus* is one of the lactic acid bacteria that is currently used in probiotic preparations. Nazef et al. showed that the majority of lactic acid bacteria found in poultry feces were *Streptococcus* and *Lactobacillus *spp. [82]. The *Ruminococcus_torques_group* has been described as bacteria that can produce SCFAs [83]. However, it has also been found that an increase in *Ruminococcus_torques_group* can disrupt the intestinal barrier and is associated with IBD and chicken tracheitis [84, 85]. Studies have reported that *Lachnospiraceae_NK4A136_group* can produce SCFAs [86]. The butyrate producing *Lachnospiraceae_NK4A136_group* can maintain the integrity of the intestinal barrier [87]. This study shows that MCE significantly increased the abundance of SCFA-producing bacteria (*Streptococcus*, *Ruminococcus_torques_group* and *Lachnospiraceae_NK4A136_group*) in the cecum of broiler chickens and significantly decreased the abundance of bacteria that are associated with colitis and steatohepatitis (*Oscillospiraceae_UCG-010*, *Barnesiella* and *Alistipes*).

### Spearman correlation analysis

By Spearman correlation analysis, this study identified *Intestinimonas*, *UCG-005*, *Barnesiella*, *Alistipes*, *RF39*, *Clostridia_UCG014*, and *Ruminococcus* as cecal bacteria that are associated with promoting inflammation and impairing growth performance. The cecal bacteria associated with anti-inflammatory and growth-promoting properties were *Anaerotruncus*, *Butyricoccus*, *Streptococcus*, *Ruminococcus_torques_group*, *Escherichia_Shigella*, *Christensenellaceae_R7_group, Negativibacillus*, *Bacteroides* and *Faecalibacterium*. Broilers with NE had significantly increased relative abundances of *Intestinimonas*, *Alistipes*, *Barnesiella*, *RF39* and *UCG-005* in the cecum, which are associated with promoting intestinal inflammation and impairing growth performance, and significantly decreased abundance of *Anaerotruncus*, which is associated with anti-inflammatory and growth-promoting performance. The relative abundances of *Anaerotruncus*, *Butyricoccus* and *Bacteroides*, which are associated with anti-inflammatory and growth-promoting properties, were significantly reduced. In contrast, MCE significantly increased the relative abundances of *Streptococcus*, *Ruminococcus_torques_group*, and *Lachnospiraceae_NK4A136_group*, which are associated with anti-inflammatory and growth-promoting properties, and significantly decreased the relative abundances of *Alistipes*, *Barnesiella* and the *UCG group*, which are associated with growth-promoting and growth-inhibiting properties. Interestingly, MCE suppressed the high abundances of *Barnesiella* and *Alistipes* under NE conditions and reversed the low abundance of *Lachnoclostridium* and *Shuttleworthia* under NE conditions. Spearman correlation analysis showed that *Barnesiella* and *Alistipes* were associated with promoting intestinal inflammation and impairing growth performance, whereas *Lachnoclostridium* and *Shuttleworthia* were associated with anti-inflammatory effects. *Lachnoclostridium* is reported to produce butyric acid, which inhibits the proliferation of pathogens and alleviates intestinal inflammation [88]. Previous studies have shown that grain feeding significantly increases the abundance of *Shuttleworthia* in the rumen, and *Shuttleworthia* is considered a butyric acid-producing microbe [89]. These results suggest that MCE can reverse the gut microbiota dysbiosis caused by NE, which in turn modulates the impact of the microbiota and its metabolites on host immune function, intestinal barrier function and growth performance and ultimately alleviates NE.

## Conclusion

NE caused intestinal inflammation, resulted in inflammatory damage, damaged the intestinal barrier, led to dysbiosis of the gut microbiota, and eventually reduced the growth performance of broilers. Dietary MCE supplementation reduced intestinal damage, improved the intestinal barrier function, enhanced immune function, suppressed excessive inflammatory damage, enriched SCFA-producing bacteria, and reduced the abundance of bacteria that are associated with inflammation and disease. These results suggest that MCE may be an effective option for the prevention of NE in poultry breeding.

### Supplementary Information

**Additional file1: Table S1.** Effects of dietary* Macleaya cordata *extract on top 10 microbes on phylum level in the cecum of broiler chickens with necrotic enteritis. **Table S2.** Effects of dietary* Macleaya cordata* extract on top 10 microbes on genus level in the cecum of broiler chickens with necrotic enteritis.

## Data Availability

The 16S rRNA gene sequencing data that were generated and analyzed during the current study are available in the NCBI primary data archive (PDA) with accession number PRJNA 936282. These data can be found here: https://www.ncbi.nlm.nih.gov/bioproject/936282.

## Abbreviations

AMPK-α1: Adenosine 5'-monophosphate (AMP)-activated protein kinase-α1
BW: Body weight
BWG: Body weight gain
ELISA: Enzyme-linked immunosorbent assay
FCR: Feed conversion ratio
FI: Feed intake
GAPDH: Glyceraldehyde-3-phosphate dehydrogenase
IFN-γ: Interferon-γ
IgA: Immunoglobulin A
IL-1β: Interleukin-1β
MCE: *Macleaya cordata* Extract
MHC-II: Major histocompatibility complex class 2
NE: Necrotic enteritis
NF-κB: Nuclear factor-κB
pIgR: Polymeric immunoglobulin receptor
RT‒PCR: Reverse transcription-polymerase chain reaction
sIgA: Secretory immunoglobulin A
TLR2: Toll-like receptor
V/C: Villus height/crypt depth
ZO-1: Zonula occludens-1
