# Supplementary material for: Dietary Macleaya cordata extract supplementation improves the growth performance and gut health of broiler chickens with necrotic enteritis
Source: J Anim Sci Biotechnol. 2023 Sep 7;14:113. doi: 10.1186/s40104-023-00916-2 (PMC10483844; doi:10.1186/s40104-023-00916-2)
Supplement: Supplementary file 1 — Additional file1: Table S1. Effects of dietary Macleaya cordata extract on top 10 microbes on phylum level in the cecum of broiler chickens with necrotic enteritis. Table S2. Effects of dietary Macleaya cordata extract on top 10 microbes on genus level in the cecum of broiler chickens with necrotic enteritis. [file 40104_2023_916_MOESM1_ESM.docx]

| **Table S1** Effects of dietary *Macleaya cordata* extract on top 10 microbes on phylum level in the cecum of broiler chickens with necrotic enteritis | | | | | | | | | | | |
| --- | --- | --- | --- | --- | --- | --- | --- | --- | --- | --- | --- |
|  |  |  |  |  |  |  |  |  |  |  |  |
| **NE** | **MCE, mg/kg** | **Firmicutes** | **Bacteroidota** | **Proteobacteria** | **Desulfobacterota** | **Actinobacteriota** | **Verrucomicrobiota** | **Deferribacterota** | **Synergistota** | **Acidobacteriota** | **Cyanobacteria** |
| - | 0 | 37.61^b^ | 56.49^ab^ | 1.02 | 1.97 | 1.04 | 0.83 | 0.25 | 0.30 | 0.15 | 0.10 |
|  | 350 | 46.41^ab^ | 45.75^bc^ | 2.90 | 2.31 | 0.44 | 0.49 | 0.42 | 0.42 | 0.00 | 0.07 |
| + | 0 | 32.02^b^ | 66.18^a^ | 0.40 | 0.78 | 0.22 | 0.16 | 0.16 | 0.01 | 0.00 | 0.03 |
|  | 350 | 56.10^a^ | 36.67^c^ | 2.12 | 1.67 | 0.82 | 1.12 | 0.79 | 0.00 | 0.08 | 0.05 |
| SEM | | 2.802 | 3.027 | 0.421 | 0.297 | 0.194 | 0.205 | 0.154 | 0.089 | 0.042 | 0.022 |
| Main-effect | | | | | | | | | | | |
| NE | - | 42.01 | 51.12 | 1.96 | 2.14 | 0.74 | 0.66 | 0.34 | 0.36 | 0.08 | 0.08 |
|  | + | 44.06 | 51.43 | 1.26 | 1.22 | 0.52 | 0.64 | 0.47 | 0.01 | 0.04 | 0.04 |
| MCE | 0 | 34.81 | 61.34 | 0.71 | 1.37 | 0.63 | 0.49 | 0.20 | 0.16 | 0.08 | 0.06 |
|  | 350 | 51.25 | 41.21 | 2.51 | 1.99 | 0.63 | 0.80 | 0.61 | 0.21 | 0.04 | 0.06 |
| *P*-value (Kruskal-wallis test) | | | | | | | | | | | |
| NE | | 0.931 | 0.603 | 0.729 | 0.057 | 0.603 | 0.563 | 0.977 | 0.073 | 0.717 | 0.466 |
| MCE | | 0.009 | 0.007 | 0.038 | 0.149 | 0.133 | 0.355 | 0.208 | 0.642 | 0.247 | 0.096 |
| *NE* Necrotic enteritis, *MCE* *Macleaya cordata* extract, *SEM* Standard error of mean  ^a-c^Different letters in the shoulder markers in the table indicate significant differences between groups (*P* < 0.05) | | | | | | | | | | | |

| **Table S2** Effects of dietary *Macleaya cordata* extract on top 10 microbes on genus level in the cecum of broiler chickens with necrotic enteritis | | | | | | | | | | | |
| --- | --- | --- | --- | --- | --- | --- | --- | --- | --- | --- | --- |
|  |  |  |  |  |  |  |  |  |  |  |  |
| **NE** | **MCE, mg/kg** | ***Bacteroides*** | ***Barnesiella*** | ***Faecalibacterium*** | ***Alistipes*** | ***Ruminococcus*** | ***Clostridia_UCG-014*** | ***Muribaculaceae*** | ***Eubacterium*** | ***Blautia*** | ***Anaerotruncus*** |
| - | 0 | 31.32^a^ | 8.07^b^ | 3.80 | 11.63^b^ | 4.16 | 2.88^b^ | 3.12 | 1.00^b^ | 0.75 | 0.27 |
|  | 350 | 15.25^b^ | 17.13^b^ | 10.43 | 8.88^b^ | 4.41 | 3.32^b^ | 2.59 | 1.17^b^ | 1.20 | 1.31 |
| + | 0 | 6.74^b^ | 32.65^a^ | 3.98 | 18.62^a^ | 2.19 | 3.97^b^ | 4.16 | 1.17^b^ | 0.60 | 0.03 |
|  | 350 | 9.75^b^ | 10.74^b^ | 7.53 | 10.47^b^ | 3.69 | 7.54^a^ | 2.42 | 2.76^a^ | 1.92 | 0.05 |
| SEM | | 2.168 | 2.271 | 1.379 | 0.867 | 0.473 | 0.394 | 0.461 | 0.231 | 0.296 | 0.251 |
| Main-effect | | | | | | | | | | | |
| NE | - | 23.29 | 12.60 | 7.11 | 10.26 | 4.29 | 3.10 | 2.85 | 1.08 | 0.97 | 0.79 |
|  | + | 8.24 | 21.70 | 5.75 | 14.55 | 2.94 | 5.75 | 3.29 | 1.96 | 1.26 | 0.04 |
| MCE | 0 | 19.03 | 20.36 | 3.89 | 15.13 | 3.18 | 3.42 | 3.64 | 1.08 | 0.68 | 0.15 |
|  | 350 | 12.50 | 13.93 | 8.98 | 9.68 | 4.05 | 5.43 | 2.51 | 1.96 | 1.56 | 0.68 |
| *P-*value (Kruskal-wallis test) | | | | | | | | | | | |
| NE | | 0.028 | 0.083 | 0.419 | 0.073 | 0.299 | 0.021 | 0.686 | 0.038 | 0.488 | 0.001 |
| MCE | | 0.204 | 0.166 | 0.024 | 0.021 | 0.149 | 0.119 | 0.175 | 0.119 | 0.057 | 0.191 |
| *NE* Necrotic enteritis, *MCE* *Macleaya cordata* extract, *SEM* Standard error of mean  ^a,b^Different letters in the shoulder markers in the table indicate significant differences between groups (*P* < 0.05) | | | | | | | | | | | |
